# Single-atom ruthenium on nitrogen-doped carbon for catalytic polysulfide conversion in lithium–sulfur batteries

**DOI:** 10.3389/fchem.2026.1921103

**Published:** 2026-09-03

**Authors:** Jiyuan Chen

**Affiliations:** School of Materials Science and Engineering, Shenzhen MSU-BIT University, Shenzhen, China

**Keywords:** lithium–sulfur batteries, nitrogen-doped carbon, polysulfide conversion, ruthenium, single-atom catalyst

## Abstract

**Background:**

Lithium, sulfur batteries are promising next-generation energy, storage systems, but their practical performance is limited by lithium polysulfide (LiPS) shuttling, sluggish sulfur redox kinetics, insulating Li_2_S formation, and performance decay under high-sulfur-loading and lean-electrolyte conditions.

**Methods:**

Single-atom Ru–NC was synthesized through precursor coordination, pyrolysis, acid washing, and secondary annealing, followed by sulfur loading through melt diffusion to obtain S/Ru–NC. The materials were characterized by microscopy, XRD, Raman spectroscopy, nitrogen sorption, XPS, ICP-OES, XANES, WT-EXAFS, and EXAFS fitting. LiPS adsorption, symmetric-cell redox kinetics, Li_2_S nucleation/decomposition, standard-loading Li–S cell performance, high-loading lean-electrolyte cell behavior, and DFT adsorption analyses were evaluated. Minimum-energy paths were further examined by climbing-image nudged elastic band calculations, and cycled high-loading cells were characterized by SEM, separator XPS, and cathode TEM.

**Results:**

Ru–NC retained a high surface area before sulfur loading and exhibited atomically dispersed Ru–N_4_ coordination with a Ru content of 0.42 wt%. S/Ru–NC contained 69.4 wt% sulfur. Compared with NC, Ru–NC increased Li_2_S_6_ adsorption efficiency from 31.6% to 78.4%, increased adsorption capacity from 0.79 to 1.96 mmol g^−1^, reduced peak-potential separation from 0.77 to 0.58 V, and lowered interfacial charge-transfer resistance from 58.7 to 31.4 Ω. Ru–NC also improved Li_2_S nucleation and decomposition, increasing Li_2_S deposition capacity from 156.8 to 286.4 mAh g^−1^ and decreasing decomposition overpotential from 232 to 148 mV. In standard-loading cells, S/Ru–NC delivered 1,276.4 mAh g^−1^ at 0.1 C and retained 612.4 mAh g^−1^ after 500 cycles at 1.0 C. Under high-loading lean-electrolyte conditions, S/Ru–NC achieved 4.84 mAh cm^−2^ initially and retained 3.56 mAh cm^−2^ after 100 cycles. DFT results confirmed stronger adsorption of sulfur species and greater charge transfer on Ru–NC. In high-loading cells cycled at 1.0 C, S/Ru–NC retained 566.3 ± 24.9 mAh g^-1^ (2.55 ± 0.12 mAh cm^−2^) after 50 cycles. The calculated rate-determining Li_2_S_2_-to-Li_2_S barrier decreased from 1.32 eV on NC to 0.68 eV on Ru–NC, while the reverse Li_2_S oxidation barrier decreased from 1.21 to 0.64 eV.

**Conclusion:**

Atomically dispersed Ru–N_4_ sites on nitrogen-doped carbon effectively couple LiPS adsorption with catalytic redox conversion, thereby improving Li_2_S reaction kinetics and Li–S cell performance under both standard and practical operating conditions. The matched reduction and oxidation barriers show that stronger binding does not create an irreversible Li_2_S trap.

## 1 Introduction

Lithium–sulfur batteries couple a high-capacity sulfur cathode with multistep solid–liquid–solid conversion chemistry, but practical operation requires simultaneous control of electronic transport, lithium-ion access, migration of lithium polysulfides (LiPSs), and Li_2_S precipitation. These processes become increasingly interdependent as sulfur loading rises and electrolyte availability decreases. A catalytic host must therefore immobilize soluble intermediates without trapping them irreversibly, accelerate both reduction and oxidation, and preserve accessible reaction pathways during extended cycling. The central scientific question is whether an atomically defined coordination site can achieve this adsorption–conversion balance under both conventional and practically constrained cell conditions (Peng et al., 2017; Seh et al., 2016; Zhou et al., 2022).

Carbonaceous sulfur hosts have been widely used to improve electrical conductivity and physically confine sulfur species. However, nonpolar carbon alone often provides insufficient chemical interaction with soluble LiPSs and limited catalytic acceleration of sulfur conversion. Structural optimization of porous carbon hosts can improve sulfur utilization, but practical performance also requires active interfacial sites that can adsorb LiPSs and promote their reversible conversion (Yang et al., 2018). Therefore, recent Li–S cathode design has shifted from passive sulfur confinement to catalytic host engineering, particularly to accelerate liquid–solid conversion processes involving Li_2_S nucleation and decomposition (Sun et al., 2018).

Single-atom catalysts provide an attractive route for regulating Li–S redox chemistry because isolated metal centers can offer high atom utilization, well-defined coordination environments, and tunable interactions with LiPS intermediates (Wang et al., 2020). In nitrogen-doped carbon matrices, M–N_x_ moieties can simultaneously increase polarity, enhance LiPS anchoring, and reduce kinetic barriers for sulfur redox reactions. Previous studies have shown that atomically dispersed Co, Fe, Sn, V, and dual-metal sites can improve LiPS adsorption, charge transfer, sulfur conversion kinetics, and cycling stability in Li–S batteries (Du et al., 2019; Li et al., 2020; Lu et al., 2020; Ma et al., 2021; Xiao et al., 2022; Yang et al., 2023). These findings indicate that the catalytic behavior of single-atom hosts is strongly dependent on the identity of the metal center, coordination structure, electronic configuration, and accessibility of the active site.

Despite substantial progress with Fe-, Co-, Ni-, Sn-, and V-based single-atom hosts, three gaps remain. First, catalyst benchmarking frequently emphasizes low-loading cells and omits electrolyte demand. Second, atomic coordination, reaction barriers, Li_2_S precipitation/oxidation, and practical-cell behavior are seldom connected within one evidence chain. Third, Ru-based single-atom hosts remain comparatively underexamined, particularly as low-metal-loading Ru–N_4_ sites in a simple nitrogen-doped carbon matrix rather than in multicomponent heterostructures (Chen et al., 2025; Du et al., 2019; Jung et al., 2022; Li et al., 2020; Li et al., 2023; Lu et al., 2020; Ma et al., 2021; Xiao et al., 2022; Yang et al., 2023).

Here, low-loading Ru–N_4_ sites on nitrogen-doped carbon are evaluated through a deliberately linked framework: atomic-structure verification, LiPS adsorption, bidirectional Li_2_S kinetics, explicit DFT reaction barriers, and cell testing under standard and high-loading lean-electrolyte conditions. The distinctive contribution is not a single peak capacity, but the experimentally and computationally consistent demonstration that isolated Ru–N_4_ sites strengthen intermediate binding while lowering the barriers for surface regeneration. This design also permits direct comparison with recent Ru-based and non-Ru single-atom catalysts using practical parameters.

- Atomically dispersed Ru–N_4_ sites were constructed on nitrogen-doped carbon nanosheets and retained after sulfur loading.
- The Ru–NC host improved Li_2_S_6_ adsorption, increased LiPS redox current response, and reduced interfacial charge-transfer resistance.
- Ru–NC promoted Li_2_S nucleation and decomposition, as shown by higher deposition/decomposition capacities and lower overpotentials.
- S/Ru–NC improved rate capability and long-term cycling stability in standard-loading Li–S cells.
- S/Ru–NC maintained higher gravimetric and areal capacities under high-sulfur-loading and lean-electrolyte conditions.
- DFT analysis linked the experimental performance improvement to stronger adsorption, shorter Ru–S/Li–N interactions, and enhanced charge transfer on Ru–NC.

## 2 Materials and methods

### 2.1 Materials

Ruthenium(III) chloride hydrate (RuCl_3_·xH_2_O), melamine, glucose, sublimed sulfur, lithium sulfide (Li_2_S), lithium bis(trifluoromethanesulfonyl)imide (LiTFSI), lithium nitrate (LiNO_3_), 1,3-dioxolane (DOL), 1,2-dimethoxyethane (DME), poly(vinylidene fluoride) (PVDF), N-methyl-2-pyrrolidone (NMP), Super P carbon, aluminum foil, lithium metal foil, and Celgard 2400 separators were used without further purification. Electrolyte preparation, LiPS solution handling, and cell assembly were conducted in an Ar-filled glovebox with O_2_ and H_2_O levels below 0.5 ppm.

### 2.2 Synthesis of single-atom Ru/N-doped carbon

Single-atom ruthenium anchored on nitrogen-doped carbon was prepared through precursor coordination, pyrolysis, acid washing, and secondary annealing. Briefly, 2.0 g of melamine and 0.5 g of glucose were dissolved in 40 mL of deionized water under continuous stirring. RuCl_3_·xH_2_O was added to obtain a nominal Ru content of 0.50 wt% relative to the expected carbon product. The solution was stirred for 6 h, freeze-dried, and ground into a uniform precursor powder. The precursor was pyrolyzed at 800 °C for 2 h under Ar at a heating rate of 5 °C min^−1^.

**FIGURE 1:**
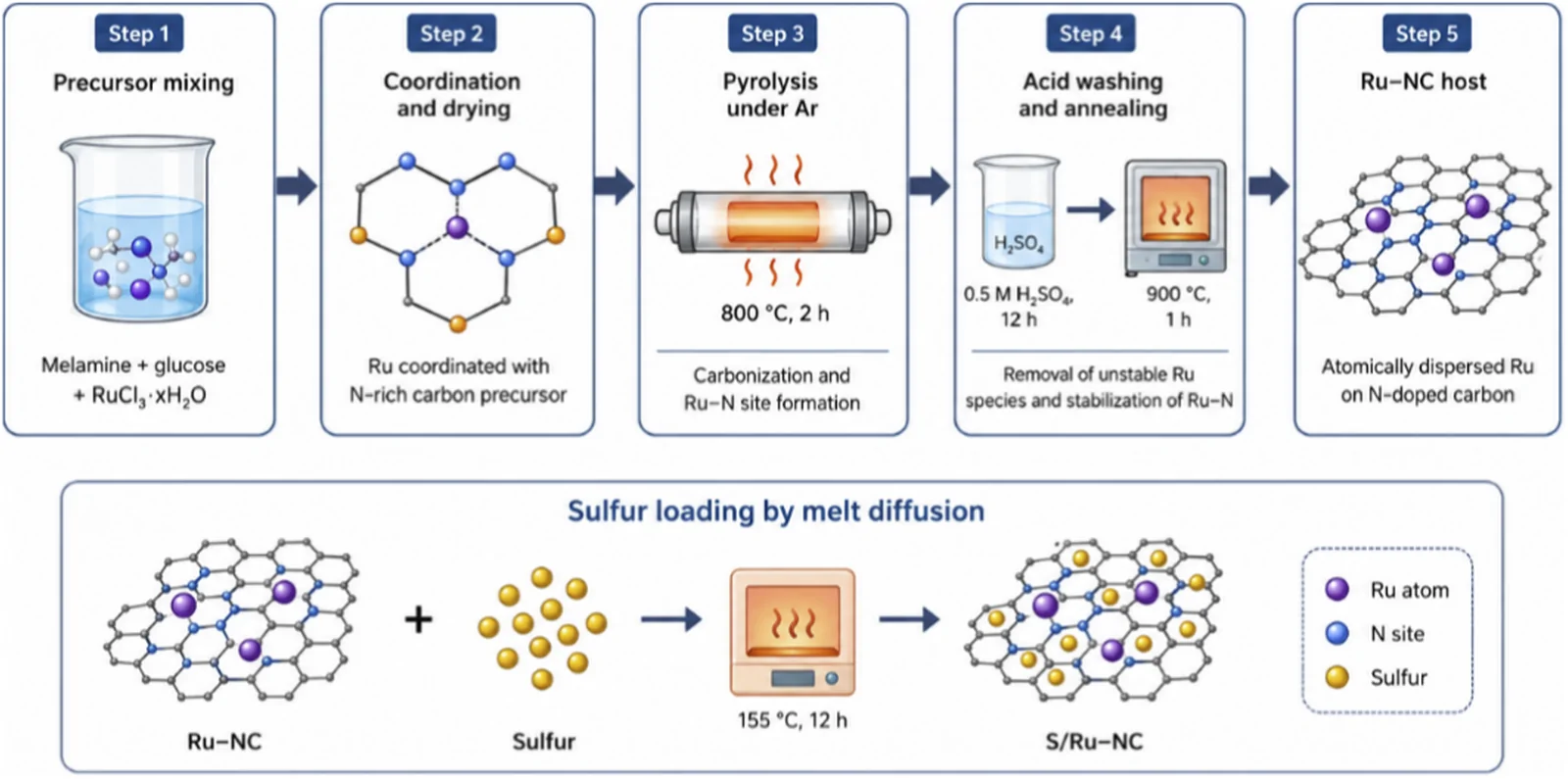
Schematic workflow of Ru–NC synthesis and sulfur loading.

The resulting black powder was washed with 0.5 M H_2_SO_4_ for 12 h to remove unstable Ru species and possible Ru nanoparticles, followed by repeated washing with deionized water and ethanol. The powder was dried at 60 °C overnight and then annealed at 900 °C for 1 h under Ar to improve carbon conductivity and stabilize Ru–N coordination. The final catalyst was denoted Ru–NC. The final Ru content was 0.42 wt%, as determined by inductively coupled plasma optical emission spectroscopy. Nitrogen-doped carbon without Ru was synthesized under identical conditions and denoted NC. The principal preparation parameters are listed in Table 1. The overall Ru–NC synthesis route and subsequent sulfur-loading process are schematically presented in Figure 1.

**TABLE 1:** Main synthesis and material parameters.

Parameter	Value or condition
Ru precursor	RuCl_3_·xH_2_O
Carbon precursor	Glucose
Nitrogen precursor	Melamine
Nominal Ru content	0.50 wt%
Final Ru content in Ru–NC	0.42 wt%
Primary pyrolysis	800 °C, 2 h, Ar
Heating rate	5 °C min^−1^
Acid washing	0.5 M H_2_SO_4_, 12 h
Secondary annealing	900 °C, 1 h, Ar
Control material	NC without Ru

### 2.3 Preparation of sulfur-loaded composites and cathodes

Sulfur-loaded Ru–NC composites were prepared by melt diffusion. Ru–NC and sublimed sulfur were mixed at a mass ratio of 30:70, ground for 30 min, sealed in a glass vial, and heated at 155 °C for 12 h. The obtained composite was denoted S/Ru–NC. The control sulfur composite, S/NC, was prepared using the same procedure. The sulfur content of S/Ru–NC was 69.4 wt%, as measured by thermogravimetric analysis under N_2_.

**FIGURE 2:**
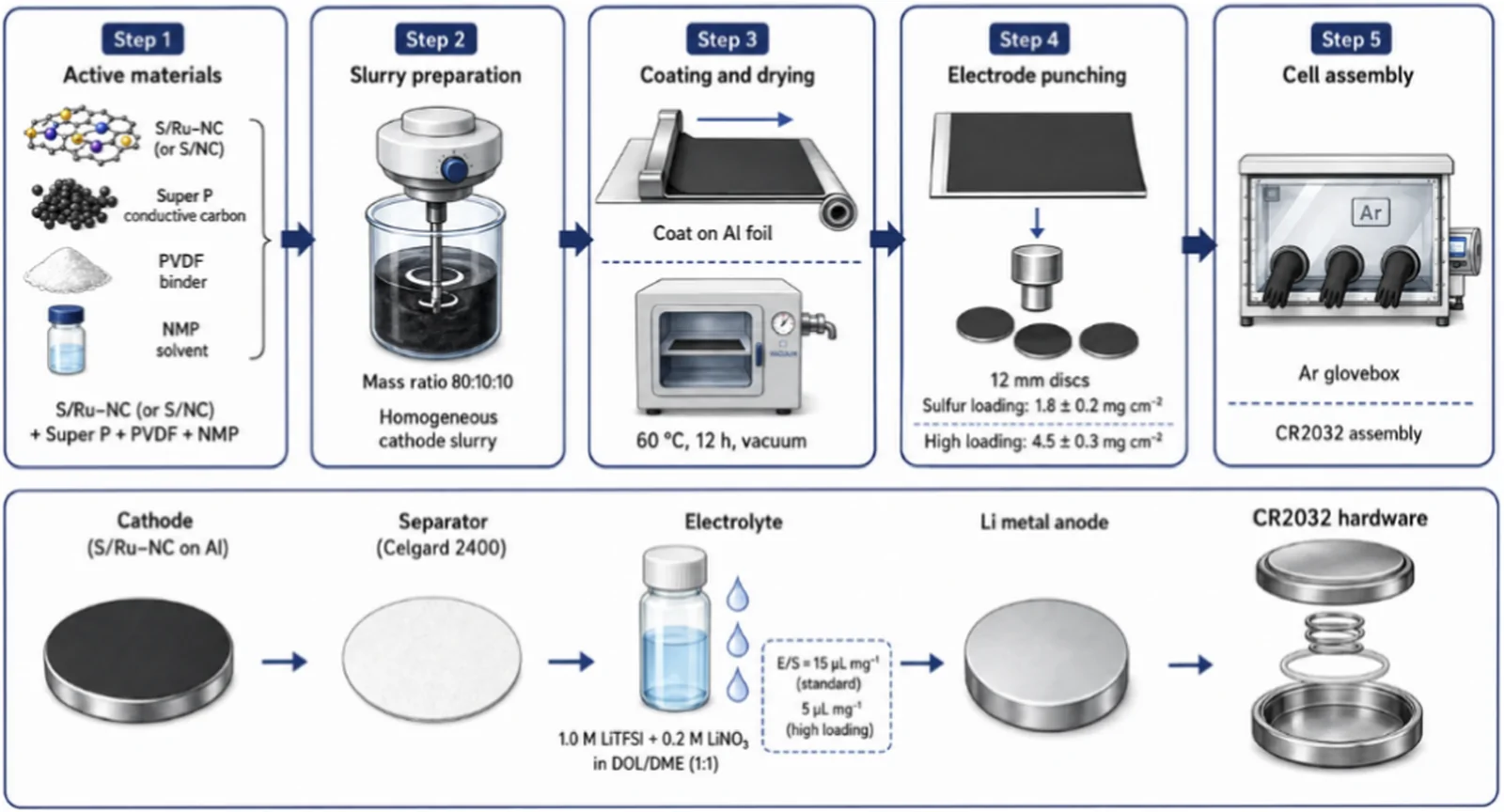
Schematic workflow of cathode fabrication and Li–S coin-cell assembly.

Cathode slurry was prepared by mixing S/Ru–NC, Super P, and PVDF at a mass ratio of 80:10:10 in NMP. The slurry was uniformly coated onto aluminum foil, dried at 60 °C for 12 h under vacuum, and punched into circular electrodes with a diameter of 12 mm. The standard sulfur loading was 1.8 ± 0.2 mg cm^−2^. High-loading cathodes were prepared with a sulfur loading of 4.5 ± 0.3 mg cm^−2^. The cathode preparation parameters are summarized in Table 2. The cathode preparation procedure and CR2032 Li–S coin-cell assembly are summarized in Figure 2.

**TABLE 2:** Cathode and cell-fabrication parameters.

Parameter	Standard-loading cells	High-loading cells
Active composite	S/Ru–NC or S/NC	S/Ru–NC or S/NC
Cathode composition	80:10:10	80:10:10
Sulfur content in S/Ru–NC	69.4 wt%	69.4 wt%
Electrode diameter	12 mm	12 mm
Sulfur loading	1.8 ± 0.2 mg cm^−2^	4.5 ± 0.3 mg cm^−2^
Electrolyte/sulfur ratio	15 μL mg^−1^	5 μL mg^−1^
Separator	Celgard 2400	Celgard 2400
Cell type	CR2032	CR2032

### 2.4 Structural and chemical characterization

The morphology and nanosheet structure of Ru–NC and S/Ru–NC were examined by scanning electron microscopy and transmission electron microscopy. High-angle annular dark-field scanning transmission electron microscopy was used to confirm the atomic dispersion of Ru. Phase structure was analyzed by X-ray diffraction using Cu Kα radiation. Raman spectroscopy was used to evaluate the degree of defects and graphitization in the carbon framework. Nitrogen adsorption–desorption analysis was performed to determine the specific surface area and pore-size distribution.

Surface elemental composition and chemical states were analyzed by X-ray photoelectron spectroscopy. The Ru loading was quantified by inductively coupled plasma optical emission spectroscopy. X-ray absorption near-edge structure and extended X-ray absorption fine structure analyses were used to verify the coordination environment of Ru and to distinguish Ru–N coordination from metallic Ru–Ru bonding.

### 2.5 LiPS adsorption and catalytic conversion tests

Li_2_S_6_ solution was prepared by dissolving Li_2_S and sulfur in a 1:5 M ratio in DOL/DME under stirring at 60 °C for 24 h. For adsorption analysis, 10 mg Ru–NC or NC was added to 5 mL of 5 mM Li_2_S_6_ solution and kept undisturbed for 12 h. The supernatant was analyzed by ultraviolet–visible spectroscopy to compare the residual LiPS concentration.

Symmetric-cell tests were conducted to evaluate the kinetics of LiPS redox conversion. Identical Ru–NC or NC electrodes were used as both working and counter electrodes. The electrolyte contained 0.2 M Li_2_S_6_ and 1.0 M LiTFSI in DOL/DME. Cyclic voltammetry was conducted between −1.0 and 1.0 V at 5 mV s^−1^. The catalytic conversion behavior was assessed from redox peak intensity, peak separation, and current response.

### 2.6 Li_2_S nucleation and decomposition tests

Li_2_S nucleation was evaluated using potentiostatic discharge. Cells were assembled with Ru–NC or NC electrodes as sulfur-host electrodes and lithium foil as the counter/reference electrode. Li_2_S_8_ catholyte was used as the sulfur source. The cells were first discharged galvanostatically to 2.06 V, then held potentiostatically at 2.05 V until the current fell below 10^–5^ A. The Li_2_S deposition capacity was calculated by integrating the current–time curve.

**FIGURE 3:**
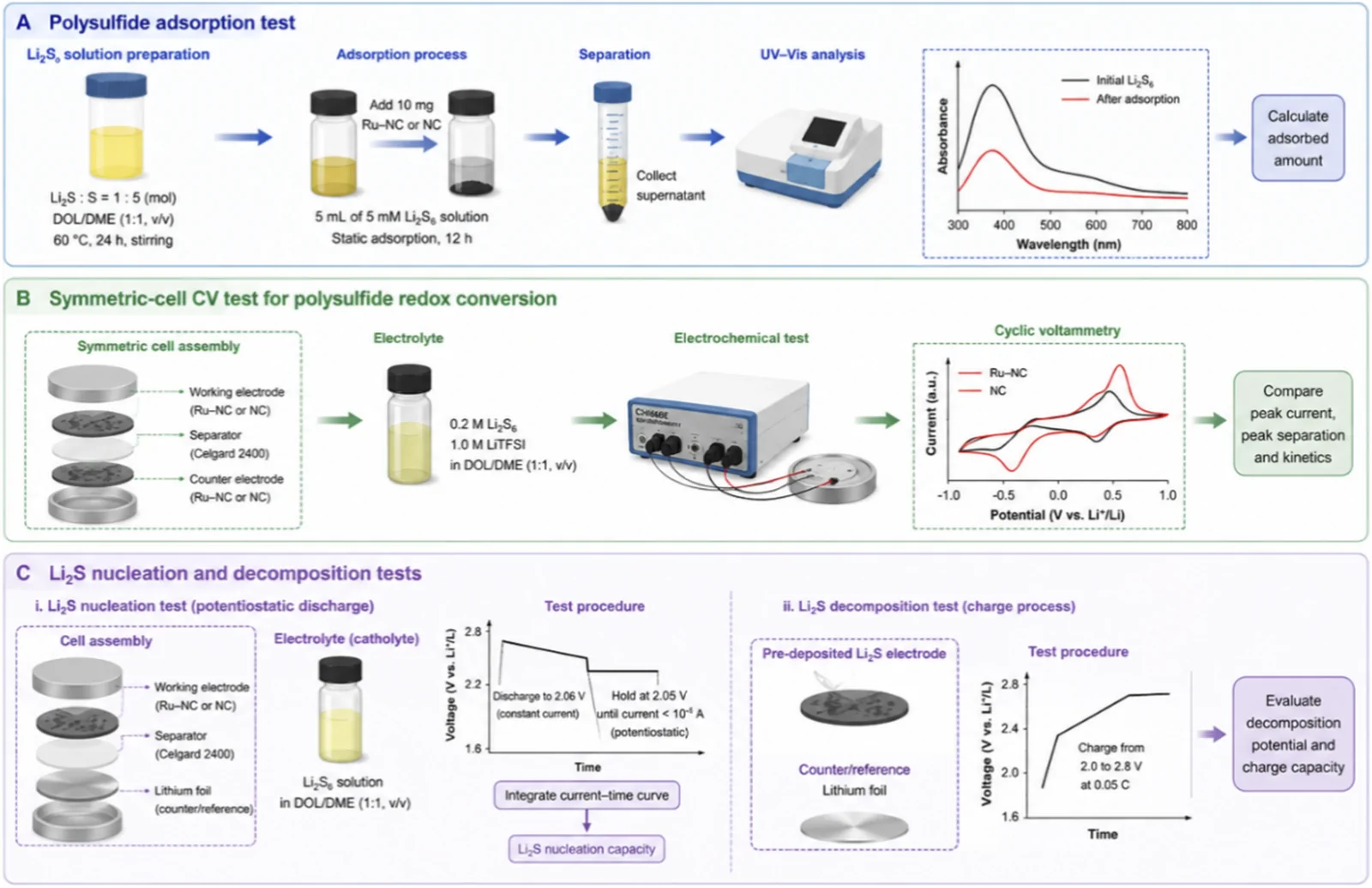
Schematic workflow of LiPS adsorption and catalytic conversion tests. **(A)** Li_2_S_6_ adsorption test, including sample preparation, adsorption, separation, UV-vis analysis, and residual-concentration evaluation. **(B)** Symmetric-cell cyclic voltammetry test for LiPS redox conversion. **(C)** Li_2_S nucleation and decomposition tests, including potentiostatic deposition and subsequent oxidation evaluation.

Li_2_S decomposition was evaluated using pre-deposited Li_2_S electrodes. The cells were charged from 2.0 to 2.8 V at 0.05 C. A lower decomposition potential and higher charge capacity indicated more favorable Li_2_S oxidation kinetics on the catalytic surface. The experimental workflow for LiPS adsorption, symmetric-cell CV, and Li_2_S nucleation/decomposition tests is shown in Figure 3.

### 2.7 Electrochemical measurements

CR2032 coin cells were assembled using S/Ru–NC cathodes, lithium metal anodes, Celgard 2400 separators, and electrolyte containing 1.0 M LiTFSI and 0.2 M LiNO_3_ in DOL/DME with a 1:1 volume ratio. The electrolyte/sulfur ratio was fixed at 15 μL mg^−1^ for standard-loading cells and 5 μL mg^−1^ for high-loading cells.

Galvanostatic charge–discharge tests were performed between 1.7 and 2.8 V versus Li^+^/Li. Rate performance was measured at 0.1, 0.2, 0.5, 1.0, and 2.0 C, followed by recovery at 0.2 C. One C was defined as 1,675 mA g^−1^ based on the theoretical capacity of sulfur. Long-term cycling was conducted at 1.0 C after two activation cycles at 0.1 C. Cyclic voltammetry was recorded from 1.7 to 2.8 V at scan rates of 0.1, 0.2, 0.5, 0.8, and 1.0 mV s^−1^. Electrochemical impedance spectroscopy was measured over the frequency range 100 kHz to 0.01 Hz with an amplitude of 5 mV. To harmonize the rate demands across configurations, an additional high-loading lean-electrolyte cohort was activated for two cycles at 0.1 C and then cycled at 1.0 C for 50 cycles (n = 3 per cathode).

### 2.8 Electrochemical calculations

All specific capacities were calculated based on the sulfur mass in the cathode. The sulfur loading was calculated using Equation 1:

$$m_{S}=\frac{m_{\text{electrode}}-m_{\text{Al}}}{A}\times w_{S}$$ (1)

where $m_{S}$ is the sulfur loading, $m_{\text{electrode}}$ is the dried electrode mass, $m_{\text{Al}}$ is the mass of the aluminum current collector, A is the electrode area, and $w_{S}$ is the sulfur mass fraction in the cathode composite. The electrolyte/sulfur ratio was calculated as Equation 2:

$$E/S=\frac{V_{\text{electrolyte}}}{m_{S,\text{total}}}$$ (2)

where $V_{\text{electrolyte}}$ is the electrolyte volume and $m_{S,\text{total}}$ is the total sulfur mass in the cathode. The specific discharge capacity was calculated as Equation 3:

$$Q=\frac{I\times t}{m_{S,\text{total}}}$$ (3)

where Q is the specific capacity, I is the applied current, t is the discharge time, and $m_{S,\text{total}}$ is the total sulfur mass. Coulombic efficiency was calculated as Equation 4:

$$CE=\frac{Q_{\text{charge}}}{Q_{\text{discharge}}}\times 100$$ (4)

Capacity retention after cycling was calculated as Equation 5:

$$R=\frac{Q_{N}}{Q_{1}}\times 100$$ (5)

where $Q_{1}$ and $Q_{N}$ are the discharge capacities of the first and Nth cycles, respectively. The peak potential separation in cyclic voltammetry was calculated as Equation 6:

$$\Delta E=E_{\text{ox}}-E_{\text{red}}$$ (6)

where $E_{\text{ox}}$ is the oxidation peak potential and $E_{\text{red}}$ is the reduction peak potential.

### 2.9 Density functional theory calculations

Density functional theory calculations were performed to examine the interaction between Ru–N_4_ sites and LiPSs. A nitrogen-doped graphene model containing a single Ru atom coordinated to four nitrogen atoms was constructed to represent the Ru–NC active site. Geometry optimization was conducted using the generalized gradient approximation with the Perdew–Burke–Ernzerhof functional. A plane-wave cutoff energy of 450 eV was used, and van der Waals interactions were included using the D3 correction. The convergence criteria for energy and force were set to 10^–5^ eV and 0.02 eV Å^−1^, respectively. The adsorption energy of Li_2_S_x_ species was calculated as Equation 7:

$$E_{\text{ads}}=E_{\text{Ru}-\text{NC}+Li_{2}S_{x}}-E_{\text{Ru}-\text{NC}}-E_{Li_{2}S_{x}}$$ (7)

where $E_{\text{Ru}-\text{NC}+Li_{2}S_{x}}$ , $E_{\text{Ru}-\text{NC}}$ , and $E_{Li_{2}S_{x}}$ are the total energies of the adsorption system, isolated Ru–NC substrate, and isolated LiPS species, respectively. A more negative adsorption energy indicated stronger interaction between the catalytic site and LiPS species. Charge-density difference and projected density-of-states analyses were used to evaluate interfacial charge redistribution and electronic coupling.

Minimum-energy pathways for Li_2_S_8_ → Li_2_S_6_ → Li_2_S_4_ → Li_2_S_2_ → Li_2_S reduction and the reverse Li_2_S oxidation step were determined using the climbing-image nudged elastic band method with five intermediate images. Electronic energies were converged to 10^–5^ eV, and the maximum residual force on each image was below 0.03 eV Å^−1^. Vibrational corrections were evaluated at 298.15 K.

The activation barrier was calculated from Equation 8:

$$E_{a}=E_{\text{TS}}-E_{\text{IS}}$$ (8)

where $E_{a}$ is the activation energy and $E_{\text{TS}}$ and $E_{\text{IS}}$ are the total energies of the transition and initial states, respectively.

The reaction free-energy change was obtained from Equation 9:

$$\Delta G={\Delta E}_{\text{DFT}}+{\Delta E}_{\text{ZPE}}-T\Delta S$$ (9)

where ΔG is the Gibbs free-energy change, ${\Delta E}_{\text{DFT}}$ is the electronic-energy difference, ${\Delta E}_{\text{ZPE}}$ is the zero-point-energy correction, T is the absolute temperature, and ΔS is the entropy change.

### 2.10 Statistical analysis

Electrochemical tests were performed using at least three independently assembled cells for each condition. Data are presented as mean ± standard deviation. Comparisons between S/Ru–NC and S/NC cells were performed using two-tailed Student’s t-tests. A value of p < 0.05 was considered statistically significant.

### 2.11 Post-cycling characterization

After 50 high-loading cycles at 1.0 C, cells were opened in the Ar-filled glovebox. Lithium anodes, separators, and cathodes were rinsed three times with anhydrous DME and vacuum-dried without air exposure. Lithium surfaces were examined by SEM and quantified for crack coverage from five fields per cell. Cathode-facing separator surfaces were analyzed by XPS, and recovered cathodes were examined by TEM/HAADF-STEM to determine Li_2_S agglomerate size and the retention of isolated Ru sites.

## 3 Results

### 3.1 Morphological, structural, textural, and sulfur-loading characteristics of Ru–NC and S/Ru–NC

Table 3 shows that Ru incorporation preserved the nanosheet geometry and most of the accessible surface and pore volume. Sulfur infusion produced the expected pore filling while retaining a mesopore-dominated transport network.

**TABLE 3:** Morphological, structural, textural, and sulfur-loading characteristics of NC, Ru–NC, and S/Ru–NC.

Parameter	NC	Ru–NC	S/Ru–NC
Nanosheet thickness, nm	3.7 ± 0.6	4.1 ± 0.7	4.6 ± 0.8
Lateral nanosheet size, μm	0.62 ± 0.15	0.66 ± 0.18	0.69 ± 0.21
BET surface area, m^2^ g^−1^	846.3	812.7	128.6
Total pore volume, cm^3^ g^−1^	1.18	1.06	0.22
Average pore diameter, nm	5.6	5.2	4.8
Micropore surface area, m^2^ g^−1^	238.4	216.9	34.7
Mesopore volume fraction, %	72.1	74.6	61.8
Graphitic interlayer spacing d002, nm	0.365	0.361	0.363
Raman D band position, cm^−1^	1,348	1,349	1,350
Raman G band position, cm^−1^	1,587	1,586	1,586
ID/IG ratio	1.08	1.14	1.13
Crystalline sulfur phase by XRD	Not detected	Not detected	Detected
Sulfur content by TGA, wt%	—	—	69.4
Residual host fraction by TGA, wt%	100.0	100.0	30.6

The modest increase in the Raman defect ratio after Ru addition is consistent with coordination-site formation, whereas the nearly unchanged graphitic spacing argues against bulk lattice distortion. The larger textural change after sulfur loading therefore reflects occupation of the host rather than structural collapse.

The combined XRD and TGA evidence confirms sulfur incorporation at 69.4 wt% and a retained carbon-host framework. This separation of catalytic-site effects from gross morphological changes is important for interpreting the electrochemical comparisons.

### 3.2 Atomic dispersion, chemical state, and coordination structure of Ru sites

As summarized in Table 4, bulk and surface analyses show that the low Ru loading was retained after sulfur incorporation. The accompanying surface-composition shift is consistent with sulfur coverage rather than loss of the active metal.

**TABLE 4:** Elemental composition, atomic dispersion, and XPS-derived chemical states of NC, Ru–NC, and S/Ru–NC.

Parameter	NC	Ru–NC	S/Ru–NC
Ru content by ICP-OES, wt%	—	0.42	0.41
Surface Ru content by XPS, at%		0.16	0.14
C content by XPS, at%	86.8	85.4	76.2
N content by XPS, at%	9.7	9.9	8.1
O content by XPS, at%	3.5	4.5	3.9
S content by XPS, at%	—	—	11.6
Mean isolated Ru-site density, atoms nm^−2^		0.91 ± 0.14	0.87 ± 0.13
Ru clusters or nanoparticles in HAADF-STEM	Not applicable	Not observed	
Ru distribution by elemental mapping	Not detected	Uniformly distributed	
Pyridinic N, % of total N	35.8	32.4	31.7
Pyrrolic N, % of total N	19.6	17.8	17.2
Graphitic N, % of total N	33.1	30.6	30.1
Oxidized N, % of total N	11.5	9.4	9.8
Ru–N contribution, % of total N	—	9.8	11.2
N 1s pyridinic-N binding energy, eV	398.4	398.5	398.5
N 1s Ru–N binding energy, eV	—	399.2	399.3
Ru 3p_3_/_2_ binding energy, eV		462.4	462.5
Ru 3p_1_/_2_ binding energy, eV		484.7	484.8
Ru chemical-state assignment		Ru–N coordination	Ru–N coordination retained

HAADF-STEM and elemental mapping resolve uniformly distributed isolated Ru sites without observable clusters. The persistent Ru–N XPS contribution after sulfur loading provides an independent chemical-state check on site retention.

The XANES, WT-EXAFS, and fitted first-shell parameters in Table 5 converge on the same structural assignment: an approximately four-coordinate Ru–N environment with no detectable Ru–Ru scattering. The agreement among complementary probes excludes metallic Ru particles as the dominant origin of activity.

**TABLE 5:** XANES, WT-EXAFS, and EXAFS fitting parameters for Ru coordination in Ru–NC and S/Ru–NC.

Sample/ref.	K-edge (eV)	WL int.	WT max. (Å^−1^)	Path	CN	R (Å)	σ^2^ (Å^2^)	Fit R	Identity
Ru–NC	22,120.1	1.31	4.6	Ru–N	4.03 ± 0.28	2.04 ± 0.02	0.0061	0.012	Atomically dispersed Ru–N_4_
S/Ru–NC	22,120.3	1.34	4.7	Ru–N	3.92 ± 0.31	2.05 ± 0.02	0.0064	0.014	Ru–N_4_ retained after sulfur loading
Ru foil	22,117.0	1.00	8.2	Ru–Ru	12.00	2.67	0.0058	0.009	Metallic Ru
RuO_2_ reference	22,121.4	1.52	5.1	Ru–O	6.00	1.98	0.0067	0.011	Ru–O coordination

### 3.3 LiPS adsorption and redox conversion kinetics

**FIGURE 4:**
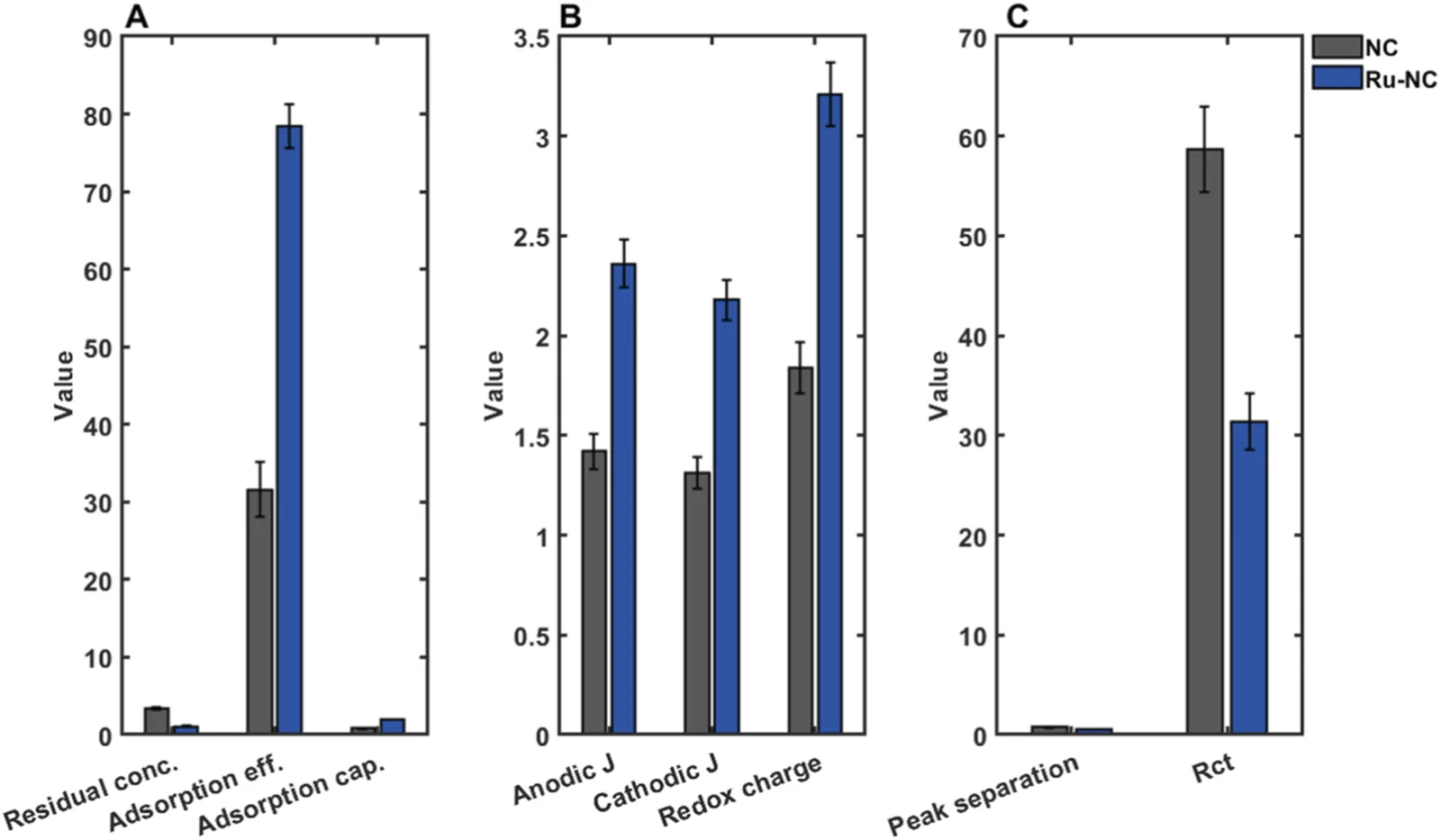
LiPS adsorption and redox-conversion kinetics of NC and Ru–NC. **(A)** Li_2_S_6_ adsorption performance, including residual Li_2_S_6_ concentration, adsorption efficiency, and adsorption capacity. **(B)** Redox activity parameters derived from symmetric-cell measurements, including anodic peak current density, cathodic peak current density, and integrated redox charge. **(C)** Kinetic descriptors, including peak-potential separation and interfacial charge-transfer resistance.

Figure 4A shows substantially greater Li_2_S_6_ removal by Ru–NC than by NC, establishing that the isolated Ru–N_4_ sites increase affinity for soluble LiPSs.

The larger symmetric-cell peak currents and integrated charge in Figure 4B demonstrate that the adsorbed intermediates remain electrochemically accessible rather than becoming inert surface deposits.

The lower peak separation and interfacial resistance in Figure 4C provide the kinetic complement to adsorption. Together, these descriptors support accelerated conversion rather than passive retention alone.

### 3.4 Li_2_S nucleation and decomposition behavior

**FIGURE 5:**
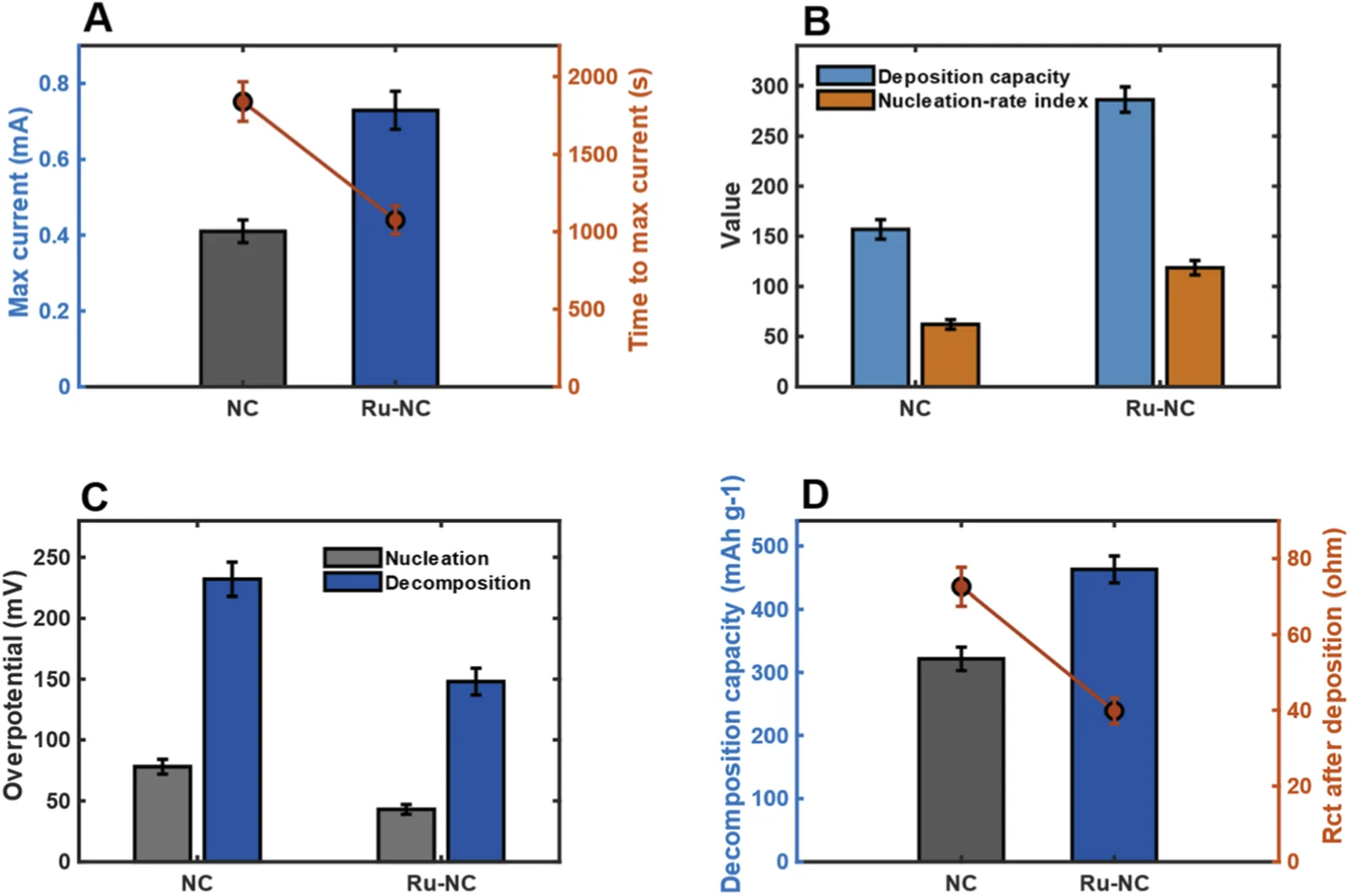
Li_2_S nucleation and decomposition kinetics on NC and Ru–NC electrodes. **(A)** Maximum Li_2_S nucleation current and time to maximum current. **(B)** Integrated Li_2_S deposition capacity and apparent nucleation-rate index. **(C)** Nucleation and decomposition overpotentials. **(D)** Li_2_S decomposition capacity and charge-transfer resistance after Li_2_S deposition.

The transient response in Figures 5A,B shows earlier Li_2_S nucleation, a larger deposition current, and greater integrated precipitation on Ru–NC. The coordinated shift in time, current, and capacity is consistent with a lower barrier for liquid-to-solid conversion.

Figure 5C shows that Ru–NC reduces both nucleation and decomposition overpotentials. The improvement in opposite reaction directions indicates bidirectional catalysis rather than a discharge-only adsorption effect.

The higher Li_2_S oxidation capacity and lower post-deposition resistance in Figure 5D further show that the deposited phase can be removed efficiently. This result directly addresses the risk that stronger adsorption might passivate the active surface.

### 3.5 Standard-loading Li–S cell performance and interfacial kinetics

**FIGURE 6:**
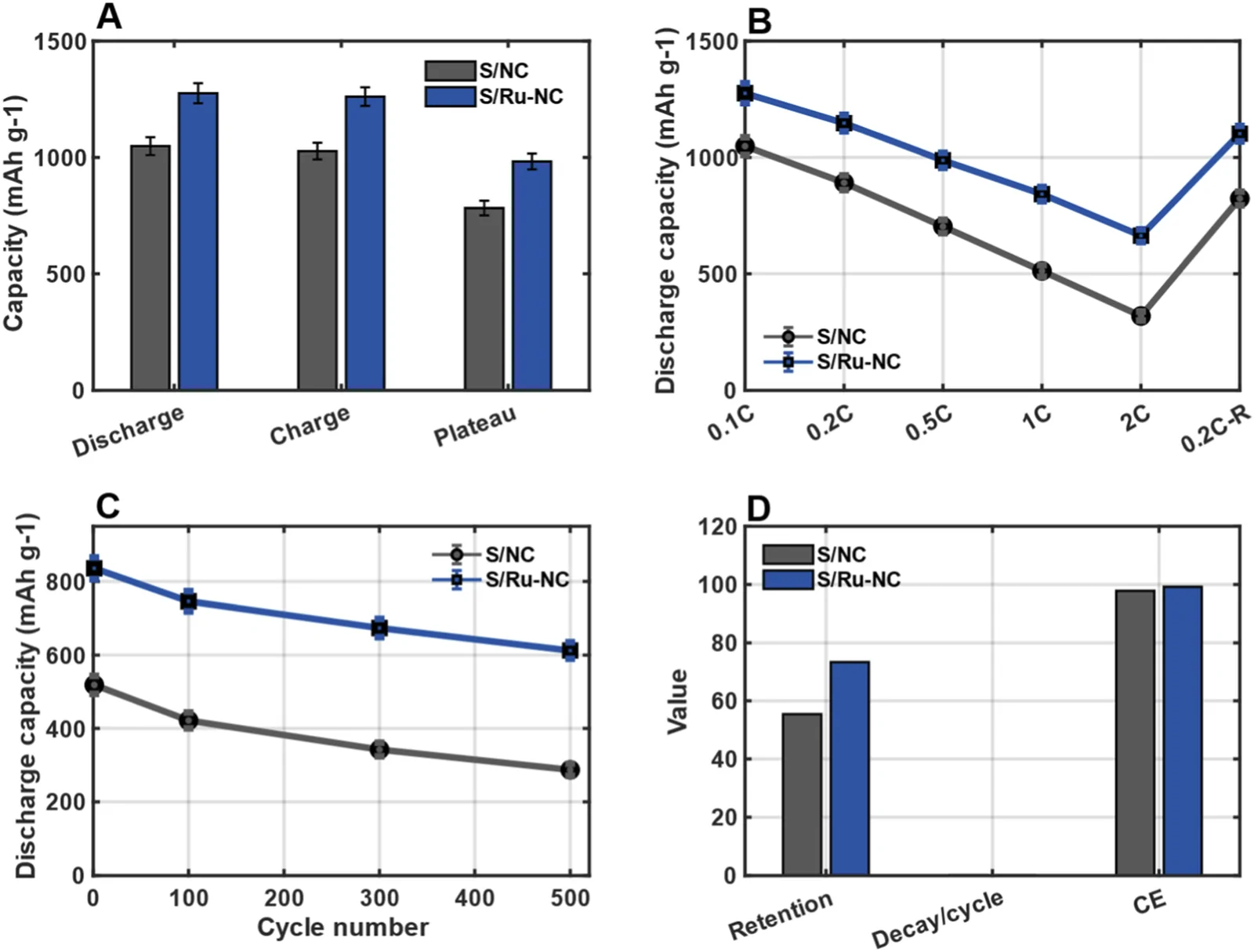
Standard-loading Li–S cell performance of S/NC and S/Ru–NC cathodes. **(A)** Initial galvanostatic capacity parameters at 0.1  C. **(B)** Rate capability from 0.1 C to 2.0  C and capacity recovery at 0.2  C. **(C)** Long-term cycling capacity at 1.0  C. **(D)** Capacity retention, average capacity decay per cycle, and average Coulombic efficiency after extended cycling.

Figure 6A shows that S/Ru–NC increased sulfur utilization and the capacity delivered on the lower-voltage discharge plateau while reducing polarization. The consistent direction across three independent cells supports a site-mediated kinetic effect.

In Figure 6B, the advantage of S/Ru–NC grows as current demand increases and remains after return to 0.2 C. This reversible rate response is consistent with faster interfacial conversion rather than irreversible activation.

Figures 6C,D show that the catalytic cathode preserves more capacity over 500 cycles, with lower per-cycle decay and Coulombic efficiency closer to unity. The sustained separation from S/NC indicates continued suppression of shuttle-related loss.

The CV and EIS descriptors in Table 6 reinforce the cycling interpretation: S/Ru–NC exhibits smaller electrochemical hysteresis and lower charge-transfer resistance both before cycling and after interphase development.

**TABLE 6:** CV and EIS-derived electrochemical parameters of standard-loading Li–S cells.

Parameter	S/NC	S/Ru–NC	Enhancement or change
First cathodic peak I, V	2.31 ± 0.01	2.34 ± 0.01	+0.03 V
First cathodic peak II, V	2.03 ± 0.02	2.07 ± 0.01	+0.04 V
Anodic peak, V	2.43 ± 0.02	2.37 ± 0.01	−0.06 V
Peak separation between anodic and cathodic II peaks, V	0.40 ± 0.03	0.30 ± 0.02	−25.0%
Cathodic peak I current at 0.1 mV s^−1^, mA	−0.34 ± 0.03	−0.51 ± 0.04	+50.0%
Cathodic peak II current at 0.1 mV s^−1^, mA	−0.48 ± 0.04	−0.76 ± 0.05	+58.3%
Anodic peak current at 0.1 mV s^−1^, mA	0.42 ± 0.03	0.69 ± 0.04	+64.3%
Fresh-cell charge-transfer resistance, Ω	119.6 ± 7.8	62.4 ± 5.1	−47.8%
Charge-transfer resistance after 50 cycles, Ω	138.2 ± 8.6	48.7 ± 4.3	−64.8%

### 3.6 High-loading lean-electrolyte Li–S cell performance

**FIGURE 7:**
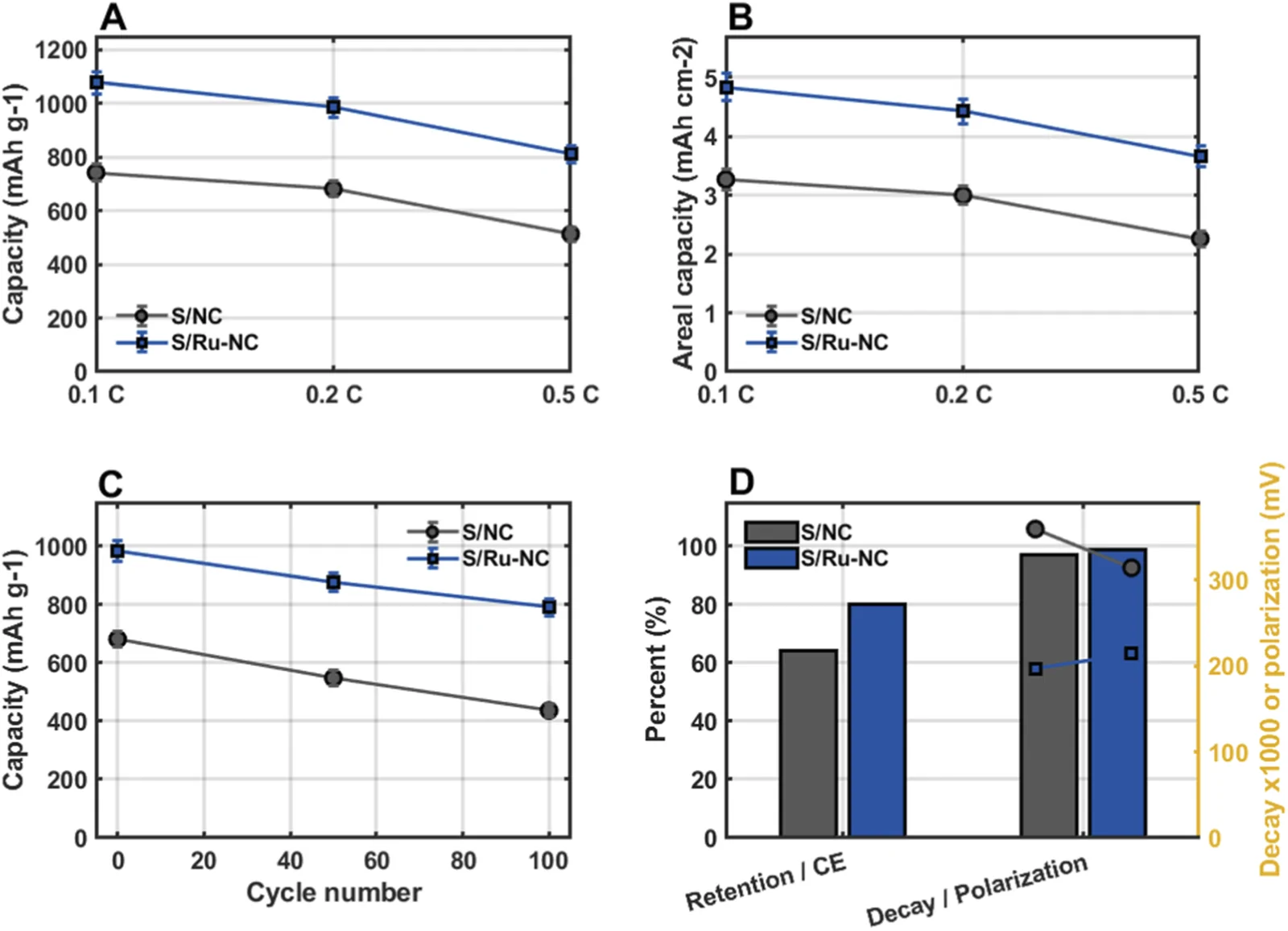
High-loading lean-electrolyte Li–S cell performance of S/NC and S/Ru–NC cathodes. **(A)** Gravimetric rate performance under high sulfur loading and lean-electrolyte conditions. **(B)** Corresponding areal rate performance. **(C)** Cycling stability at 0.2  C. **(D)** Capacity retention, average capacity decay per cycle, and charge–discharge polarization.

Under high loading and an electrolyte/sulfur ratio of 5 μL mg^−1^, Figure 7A shows that S/Ru–NC retained its gravimetric-capacity advantage throughout the tested rate sequence.

The same trend appears on an areal basis in Figure 7B. The initial areal capacity reached 4.84 ± 0.24 mAh cm^−2^, confirming that the benefit was not an artefact of normalizing by a low sulfur mass.

Figures 7C,D show continued separation during 100 cycles at 0.2 C; S/Ru–NC retained 3.56 ± 0.17 mAh cm^-2^ and exhibited lower polarization and slower capacity decay. These are coin-cell results, but they establish activity under simultaneously increased sulfur loading and restricted electrolyte.

**FIGURE 8:**
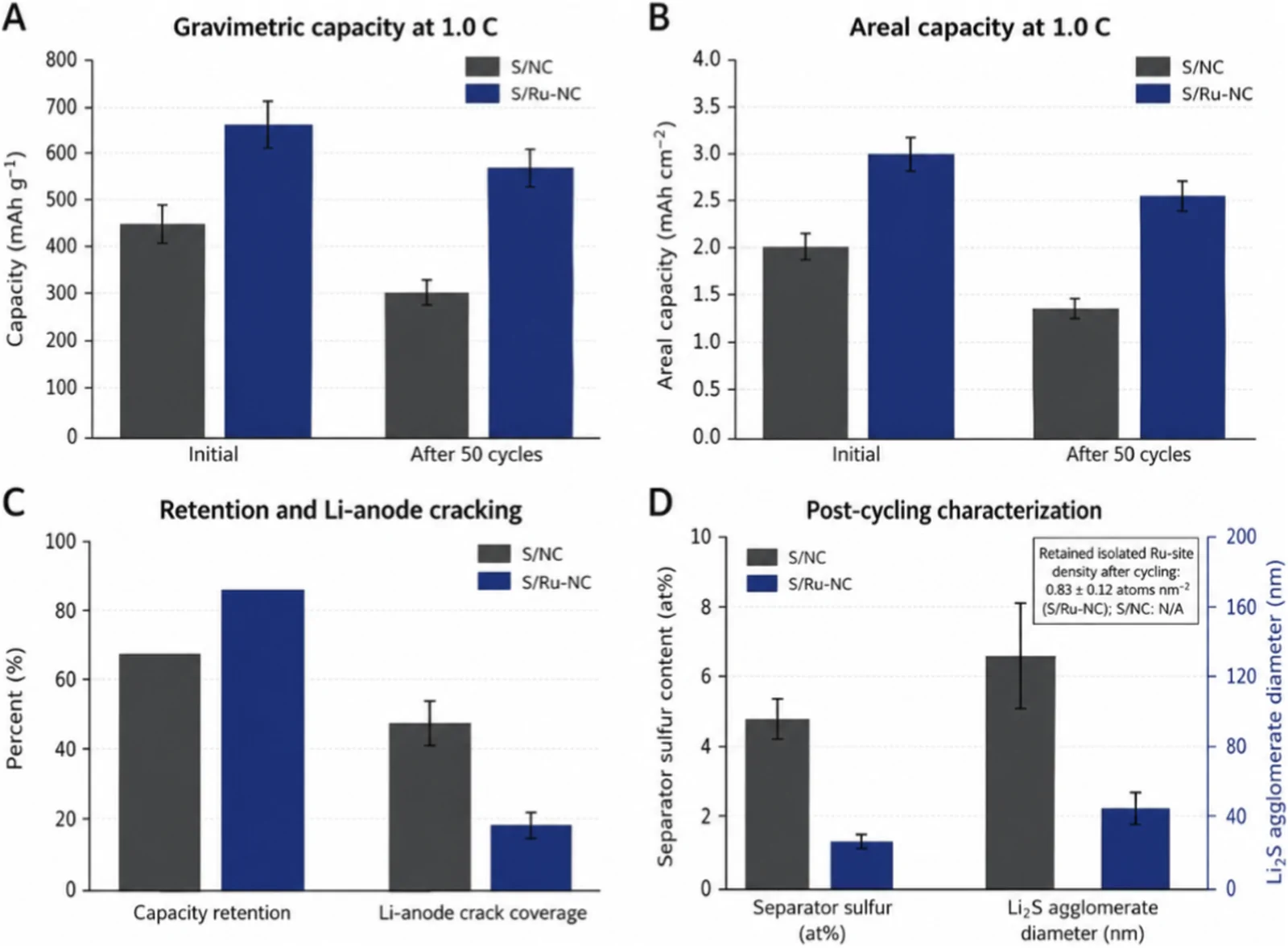
High-rate cycling and post-cycling characterization under high-loading lean-electrolyte conditions. **(A)** Gravimetric capacity of S/NC and S/Ru-NC at 1.0 C initially and after 50 cycles. **(B)** Areal capacity at 1.0 C initially and after 50 cycles. **(C)** Capacity retention and lithium-anode crack coverage after 50 cycles. **(D)** Post-cycling separator sulfur content, Li2S agglomerate diameter, and retained isolated Ru-site density.

To align the rate demand with the standard-loading protocol, high-loading cells were additionally cycled at 1.0  C. As summarized in Table 7 and Figure 8, S/Ru–NC retained higher gravimetric and areal capacities through 50 cycles and exhibited markedly lower evidence of shuttle-derived contamination after disassembly.

**TABLE 7:** High-rate cycling and post-cycling characterization of high-loading lean-electrolyte cells.

Endpoint	Unit	S/NC	S/Ru–NC
Initial discharge capacity at 1.0 C	mAh g^−1^	446.2 ± 23.8	664.1 ± 27.6
Discharge capacity after 50 cycles	mAh g^−1^	301.1 ± 18.7	566.3 ± 24.9
Initial areal capacity at 1.0 C	mAh cm^−2^	2.01 ± 0.12	2.99 ± 0.14
Areal capacity after 50 cycles	mAh cm^−2^	1.35 ± 0.09	2.55 ± 0.12
Capacity retention after 50 cycles	%	67.5	85.3
Lithium-anode crack coverage		47.8 ± 5.6	18.6 ± 3.2
Separator sulfur content by XPS	at%	4.8 ± 0.5	1.3 ± 0.2
Li_2_S agglomerate diameter by TEM	nm	126 ± 28	43 ± 11
Isolated Ru-site density after cycling	atoms nm^−2^	Not applicable	0.83 ± 0.12

Post-cycling SEM showed a smoother and less cracked lithium surface opposite S/Ru–NC, while separator XPS detected less retained sulfur. TEM of the recovered cathode showed smaller Li_2_S agglomerates and HAADF-STEM confirmed that the isolated Ru-site density remained close to its pristine value. These observations directly connect the improved cycling response with reduced LiPS crossover and reversible Li_2_S growth.

### 3.7 DFT-derived LiPS adsorption geometry and charge transfer

**FIGURE 9:**
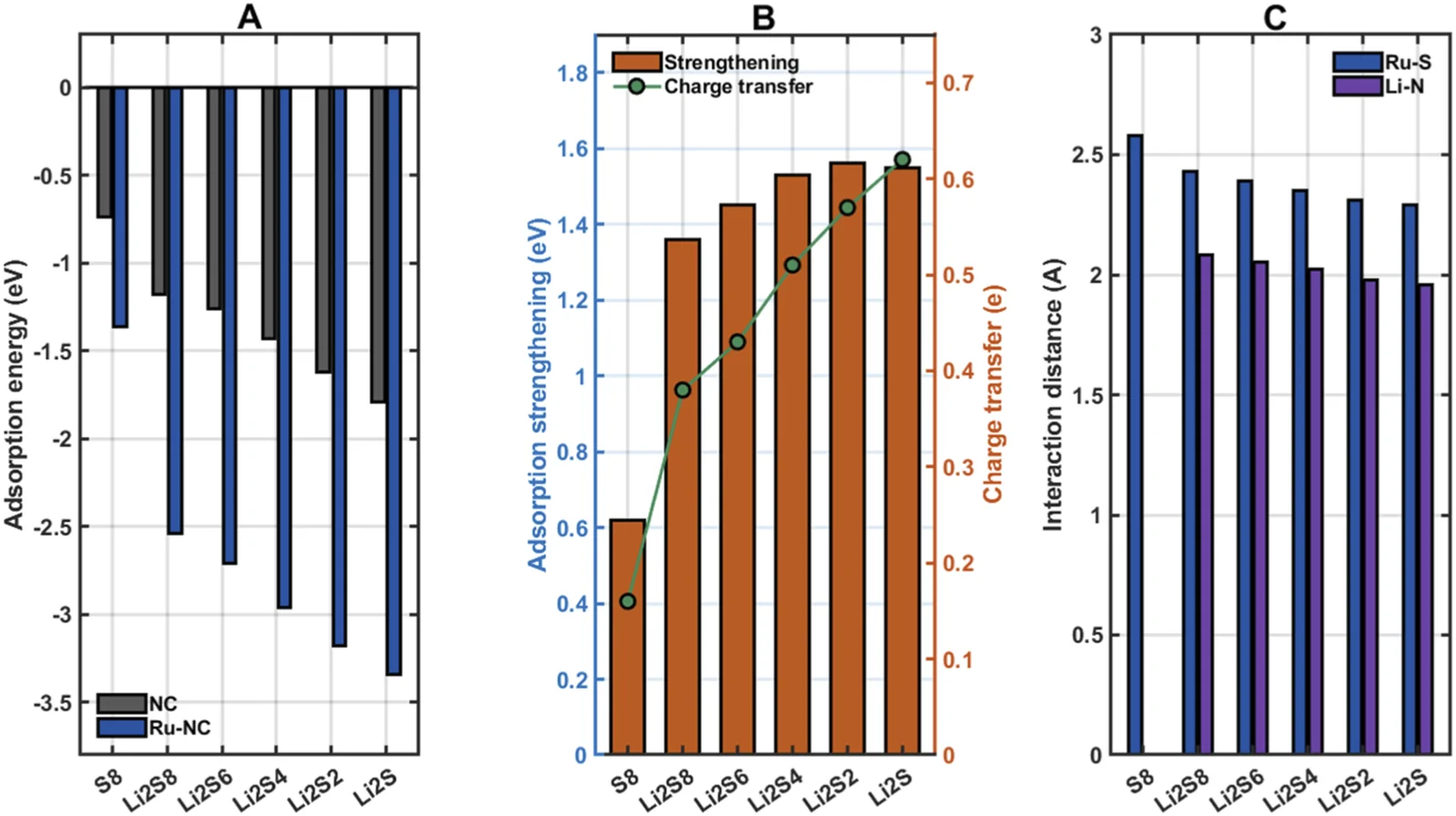
DFT-derived adsorption energetics and interfacial interactions between sulfur species and Ru–NC. **(A)** Adsorption energies of sulfur species on NC and Ru–NC. **(B)** Adsorption strengthening and charge transfer induced by Ru–N_4_ sites. **(C)** Optimized Ru–S and Li–N interaction distances.

Figure 9A shows more favorable adsorption of every modeled sulfur species on Ru–NC than on NC. The larger stabilization of lithiated intermediates than of S_8_ indicates selective polar interaction rather than nonspecific sulfur physisorption.

The charge-transfer and geometry descriptors in Figures 9B,C evolve coherently with chain shortening: electronic redistribution increases while the Ru–S and Li–N contacts contract. This trend identifies coupled metal–sulfur and nitrogen–lithium interactions at the Ru–N_4_ pocket.

Adsorption energies alone cannot distinguish catalysis from surface poisoning; the explicit transition-state analysis below therefore tests whether the stabilized intermediates remain kinetically convertible.

**FIGURE 10:**
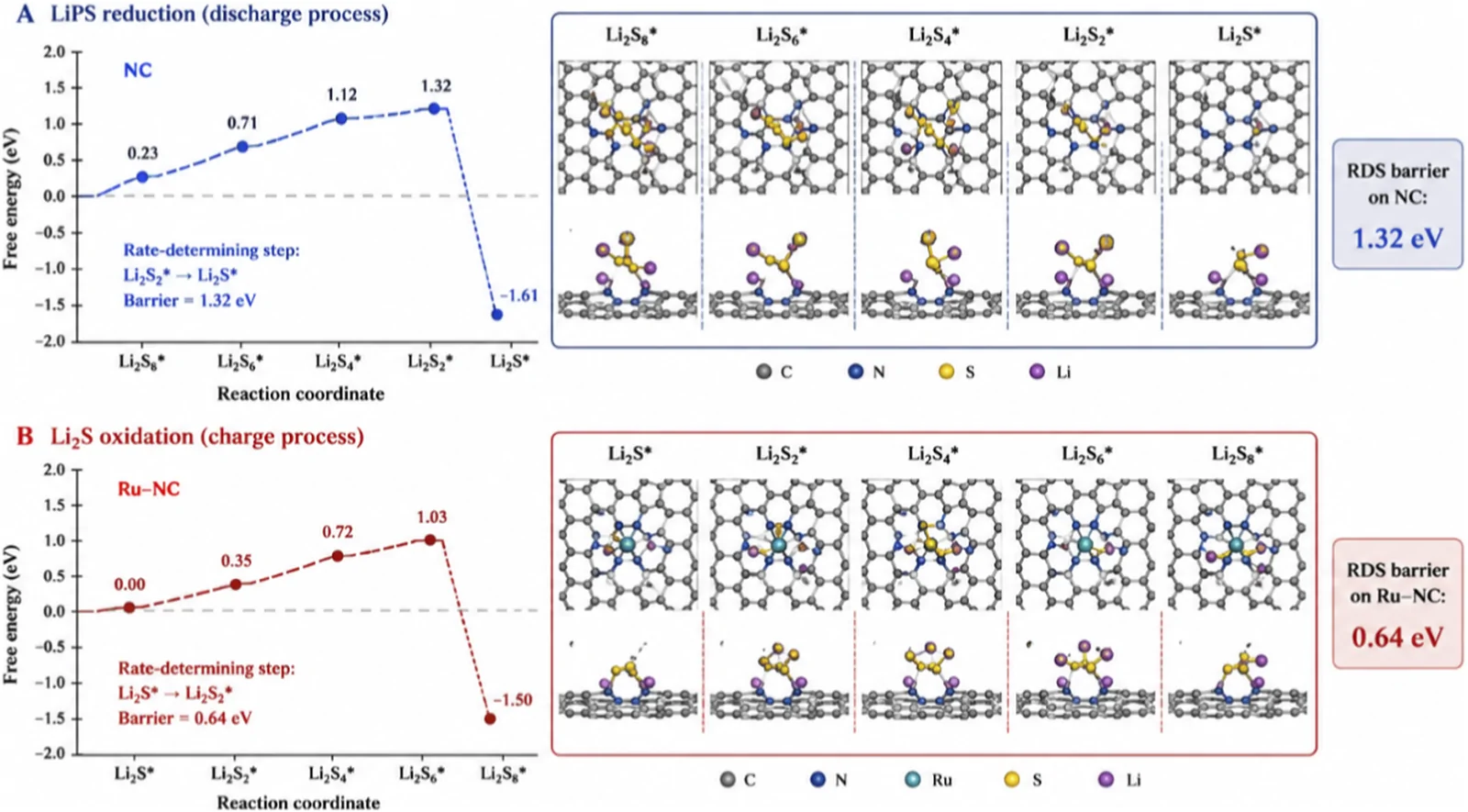
DFT reaction-energy profiles and transition-state barriers for stepwise LiPS reduction and Li_2_S oxidation on NC and Ru-NC. **(A)** LiPS reduction (discharge) pathway, including the reaction-energy profile, intermediate structures, and the rate-determining reduction barrier. **(B)** Li_2_S oxidation (charge) pathway, including the reaction-energy profile, intermediate structures, and the rate-determining oxidation barrier.

The minimum-energy paths in Figure 10 resolve the adsorption–catalysis distinction. The rate-determining Li_2_S_2_-to-Li_2_S reduction barrier decreased from 1.32 eV on NC to 0.68 eV on Ru–NC, and the maximum uphill free-energy change decreased from 1.08 to 0.46 eV. For the reverse reaction, the Li_2_S oxidation barrier decreased from 1.21 to 0.64 eV. Thus, Ru–N_4_ stabilizes polar intermediates without creating a deep terminal-state trap, consistent with the experimentally improved Li_2_S nucleation and decomposition.

## 4 Discussion

The evidence supports a structure–function sequence rather than a simple surface-area explanation. Ru incorporation preserves the porous nanosheet framework, whereas sulfur loading occupies the pores without erasing the mesoporous transport network. The catalytic comparison is therefore made between hosts of similar morphology and sulfur content, allowing the isolated coordination site to remain the principal controlled difference (Liu et al., 2018).

The active-site assignment is supported by mutually independent measurements. Microscopy excludes visible Ru aggregates, XPS identifies persistent Ru–N bonding, and X-ray absorption resolves a first-shell coordination number close to four without a Ru–Ru path. This convergence is essential because nanoparticle and single-atom Ru would present different electronic structures. The Ru–N_4_ architecture extends established M–N_x_ design principles (Du et al., 2019; Ma et al., 2021) while using a low Ru loading and a chemically simple carbon support.

Ru–N_4_ improves both capture and turnover of LiPS intermediates. Stronger solution depletion alone could indicate overbinding, but the larger symmetric-cell charge, lower peak separation, and lower interfacial resistance show that adsorbed species remain reactive (Jung et al., 2022; Xiao et al., 2022; Zhao et al., 2021). The transition-state calculations make this distinction explicit: the terminal reduction and reverse oxidation barriers both decrease on Ru–NC. Stronger adsorption is therefore paired with faster surface regeneration rather than with persistent poisoning.

The Li_2_S experiments identify the physical step affected by this balance. Earlier nucleation and more distributed precipitation limit the formation of large insulating deposits, while the lower oxidation barrier and resistance facilitate their subsequent removal. The TEM-derived decrease in agglomerate size after cycling is consistent with this mechanism. These observations agree with the broader view that reversible Li_2_S precipitation, rather than adsorption alone, controls accessible sulfur utilization (Ren et al., 2022).

At standard loading, the catalyst advantage increases with current rate and remains during capacity recovery, indicating a kinetic origin. The lower electrochemical hysteresis and resistance also persist after cycling, while the reduced capacity-decay rate suggests that faster conversion decreases the residence time of soluble intermediates. The resulting stability is consistent with the adsorption–conversion balance reported for complementary single- and dual-site catalysts (Yang et al., 2023).

The practical tests probe whether the same mechanism survives under restricted electrolyte and increased sulfur mass. The retained areal capacity, the additional 1.0 C cohort, and the post-cycling evidence all favor S/Ru–NC. Reduced sulfur accumulation on the separator and less damage to the opposing lithium surface directly support shuttle mitigation, while retained isolated Ru sites indicate catalyst durability. Nevertheless, the evidence remains limited to coin cells; pouch cells, lower lithium excess, and still lower electrolyte/sulfur ratios are required before cell-level energy density can be claimed (Li et al., 2023; Lu et al., 2020).

At the electronic-structure level, the Ru center interacts with sulfur while adjacent nitrogen atoms coordinate lithium, creating a cooperative Lewis acid–base pocket. Chain shortening strengthens charge redistribution, yet the calculated barrier maximum falls rather than rises. This response is consistent with descriptor-based studies in which orbital overlap and band-center regulation tune intermediate binding across sulfur and other conversion reactions (Chen et al., 2025).

The broader relevance of this interpretation is supported by recent studies of s–p overlap in sulfur conversion, graphene-supported atomically dispersed metals, orbital-hybridization control in reversible conversion storage, and p-band regulation in metal–gas batteries (Chen et al., 2022; Qi et al., 2025; Wang et al., 2024; Wu et al., 2025). Although the chemistries differ, these reports collectively emphasize that electronic coupling must be evaluated together with adsorption energy when identifying catalytic descriptors.

The cross-study comparison in Table 8 includes a recent Ru single-atom sulfur host (Bai et al., 2024) and representative non-Ru single-atom systems. Ru–NC combines a competitive areal capacity with an explicitly lean electrolyte, while the added high-loading 1.0 C test addresses a protocol dimension often omitted from practical comparisons. Because electrode composition, lithium excess, and activation procedures vary, the table is intended as contextual benchmarking rather than a strict performance ranking.

**TABLE 8:** Comparison with representative Ru-based and other single-atom catalysts for Li–S batteries.

Catalyst/host	Year	Sulfur loading (mg cm^−2^)	E/S (μL mg^−1^)	Initial areal capacity (mAh cm^−2^)	High-rate performance	Cycling stability
Co–N/graphene (Du et al., 2019)	2019	2.5	NR	3.00	618 mAh g^−1^ at 2 C	74% after 200 cycles at 1 C
SAFe@g-C_3_N_4_ (Lu et al., 2020)	2020	2.3	3.8	3.17	704 mAh g^−1^ at 5 C	90% after 200 cycles at 0.2 C
Fe–N coordination (Ma et al., 2021)	2021	3.2	8.0	3.82	643 mAh g^−1^ at 2 C	78% after 300 cycles at 1 C
Fe–N_4_/Ni–N_4_ (Yang et al., 2023)	2023	4.0	6.0	4.36	701 mAh g^−1^ at 2 C	81% after 200 cycles at 0.5 C
N_2_–Fe–B_2_ (Li et al., 2023)	2023	6.0	5.0	6.18	566 mAh g^−1^ at 2 C	82% after 100 cycles at 0.2 C
Ru–MoS_2_/MXene (Bai et al., 2024)	2024	1.5	NR	1.92	684.3 mAh g^−1^ at 6 C	696 mAh g^−1^ after 1,000 cycles at 2 C
V single-atom host (Chen et al., 2025)	2025	5.0	5.0	5.26	692 mAh g^−1^ at 2 C	80% after 150 cycles at 0.5 C
Ru–NC (this work)	2026	4.5	5.0	4.84	664.1 mAh g^−1^ at 1 C (high loading)	85.3% after 50 cycles at 1 C; 3.56 mAh cm^−2^ after 100 cycles at 0.2 C

**FIGURE 11:**
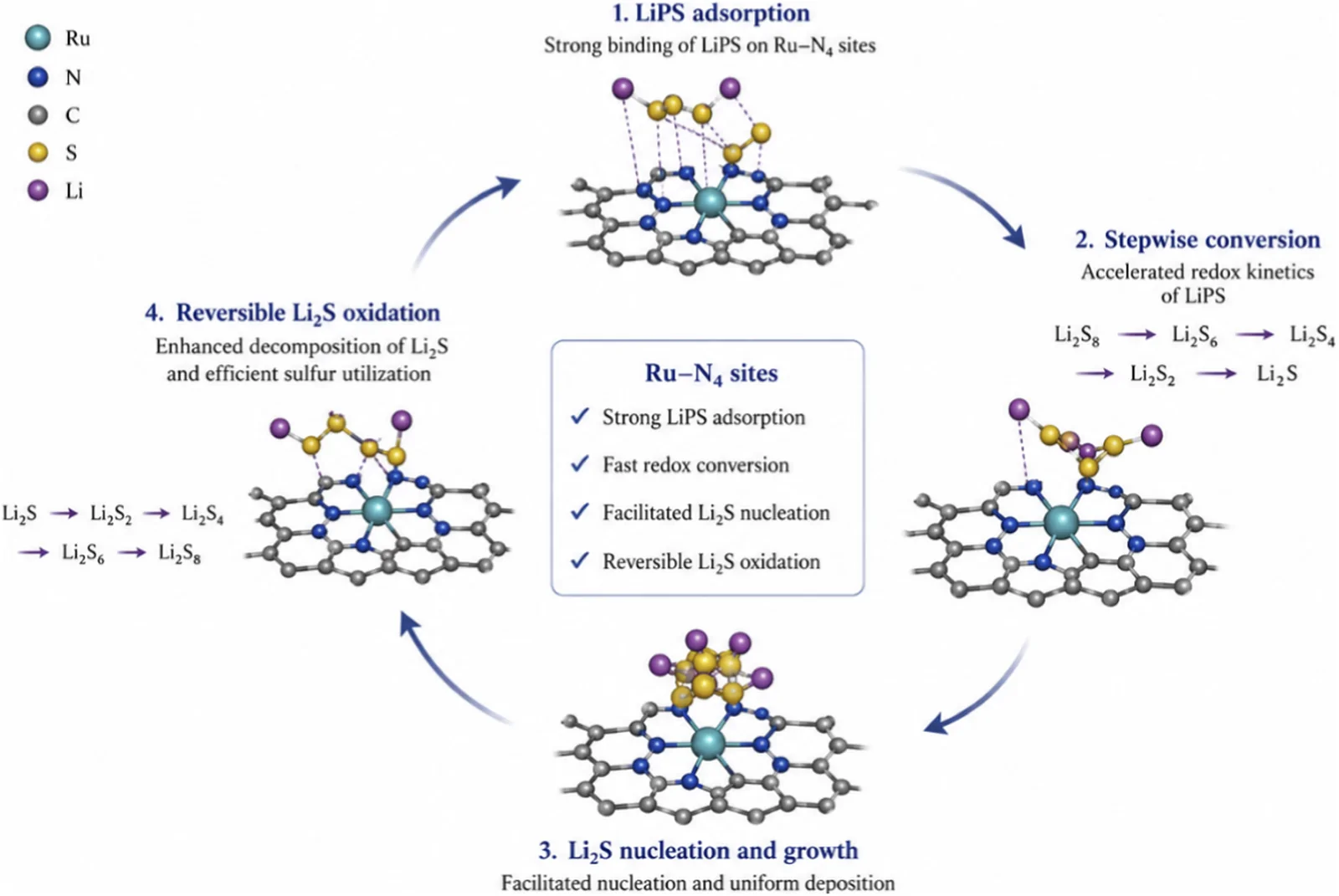
Proposed Ru–N_4_-mediated mechanism for LiPS adsorption, stepwise conversion, Li_2_S nucleation, and reversible Li_2_S oxidation.

The integrated catalytic mechanism is summarized in Figure 11. LiPSs are first polarized through cooperative Ru–S and Li–N interactions at the Ru–N_4_ pocket. Electronic coupling then lowers the barrier for chain shortening and distributes Li_2_S nucleation, while the reduced reverse barrier enables Li_2_S oxidation and active-site regeneration. The resulting shorter lifetime of soluble intermediates reduces crossover to the separator and lithium anode.

Overall, the revised evidence chain links isolated Ru–N_4_ coordination to selective LiPS binding, reduced bidirectional reaction barriers, regulated Li_2_S precipitation/oxidation, lower shuttle-derived contamination, and improved cycling under two loading regimes. The principal advance is the demonstrated balance between adsorption and turnover, rather than adsorption strength alone. The practical data are encouraging but should be interpreted as a validated coin-cell benchmark rather than as proof of commercial readiness.

## Data Availability

The original contributions presented in the study are included in the article/supplementary material, further inquiries can be directed to the corresponding author.
